# Research on Self-Aligning Flanges Based on Piezoelectric Actuators Applied to Precision Grinding Machines

**DOI:** 10.3390/mi12111393

**Published:** 2021-11-13

**Authors:** Xuepeng Huang, Zhenzhong Wang, Bingyi Shen, Pengli Lei

**Affiliations:** 1 Department of Mechanical and Electrical Engineering, Xiamen University, Xiamen 361005, China; 19920210156226@stu.xmu.edu.cn (X.H.); 19920191151158@stu.xmu.edu.cn (B.S.); 19920190154065@stu.xmu.edu.cn (P.L.); 2 Shenzhen Research Institute of Xiamen University, Shenzhen 518057, China

**Keywords:** reversal method, eccentricity, piezoelectric actuator, flange

## Abstract

Laser fusion research requires a large number of high-precision large-diameter aspherical components. To improve the grinding efficiency in the component production process, the manual operation time during the grinding process needs to be reduced. The grinding process requires the installation of the dressed grinding wheel onto the grinding machine spindle, and the off-line dressing results in installation errors during the loading and unloading process, which requires more time for manual alignment. To achieve self-aligning, the circumferential contour of the grinding wheel was first restored by the reversal method, then noise reduction and circle fitting were performed to obtain the eccentricity value and eccentricity position between the flange and the spindle, and finally, the flange was adjusted finely by three piezoelectric actuators installed inside the flange to reduce the eccentricity. Three repetitive experiments were conducted to verify that the self-aligning flange can reduce the eccentricity value by retracting the piezoelectric actuators so that the proper alignment between the flange and the spindle could meet the requirements; the average eccentricity value of the three experiments decreased by 74%, which greatly improved the efficiency of the grinding wheel alignment.

## 1. Introduction

As the precision manufacturing industry requires higher and higher surface processing accuracy for optical components [1,2], the requirements for the accuracy of the grinding process for optical components are also increasing; however, the grinding wheel mounting error has become an important concern in the high-precision grinding process. In the grinding process, the high hardness and low fracture toughness make the cutting force fluctuate greatly during grinding, which affects the surface quality of the components. Therefore, if there is a large eccentricity error in the grinding wheel installation, it will lead to an increase in the fluctuation of grinding force and affect the machining quality [3,4]. The causes of grinding wheel mounting errors are as follows: roundness error of the grinding wheel and coaxiality error of the grinding machine spindle, both of which are generally small. The important influencing factor is the eccentricity error generated by the mounting eccentricity between the grinding wheel center and the spindle axis. The installation eccentricity of the grinding wheel will directly cause changes in the thickness of the surface of the machined component, due to the unevenness of the thickness of the machined surface. Therefore, it will indirectly lead to uneven changes in the force on the grinding machine spindle, and even produce a certain impact that will shorten the working life of the grinding machine spindle [5]. Therefore, the precision mounting of grinding wheels has become one of the key problems that need to be solved in the field of precision grinding at present [6].

The process of manual precision assembly and calibration of grinding wheels is usually used to detect the circumferential runout of the grinding wheel, roughly determine the location of the eccentricity, fine-tune it by tapping the grinding wheel, and then repeat the detection of circumferential runout—and if it is not satisfied, to repeat the alignment until the circumferential runout is reduced to the qualified standard. The centering process is tedious and inefficient, so it is necessary to propose a self-aligning principle and corresponding instrument to replace manual centering, to improve the accuracy and efficiency of centering.

To achieve automatic centering, it is necessary to obtain the grinding wheel contour first, and there are mainly two methods: the machine vision method [7,8] and the reversal method [9], among which the reduction accuracy of the reversal method is higher than that that of machine vision method, and the accuracy is micron level, so the reversal method based on a laser displacement sensor for grinding wheel contour reduction is the more commonly used method for grinding wheel contour reduction at present.

Chen et al. [10] designed a grinding wheel centering device based on the reversal method to reduce the grinding wheel contour, which can effectively achieve centering by applying micro-displacement on the sidewall of the cup grinding wheel. This is the only study that uses piezoelectric actuators to achieve automatic grinding wheel aligning. However, the disassembly and installation of the centering fixture are tedious, and it can only center small-sized cup grinding wheels, which cannot be applied to most grinding applications. In order to automatically align grinding wheels of different sizes and types, this paper designs a self-aligning flange based on the commonly used flange size for mounting grinding wheels, and realizes grinding wheel alignment through a drive mechanism and a micro-displacement mechanism installed inside the flange. The main research contents of this paper are: (1) Error analysis of the inverse rotation method for reducing the grinding wheel contour. (2) Designing the self-aligning flange mechanism based on the commonly used flange dimensions. (3) The moving average filter is used to reduce the noise of the grinding wheel contour data, and the least squares method is used to fit a circle to the contour data to obtain the grinding wheel eccentricity information.

## 2. Derivation of the Self-Aligning Principle

The first step to realizing the self-aligning of the grinding wheel is to identify the outer contour of the grinding wheel and fit the optimal circular contour. According to the fitted circle, the diameter of the grinding wheel and the coordinates of the center of the circle can be obtained. At present, the commonly used methods to restore the circle contour include the machine vision contour restoration method and the reversal method based on the laser displacement sensor. Since the grinding wheel of a precision grinder needs high-precision restoration, and the machine vision restoration accuracy is low, the reversal method is adopted.

**Figure 1:**
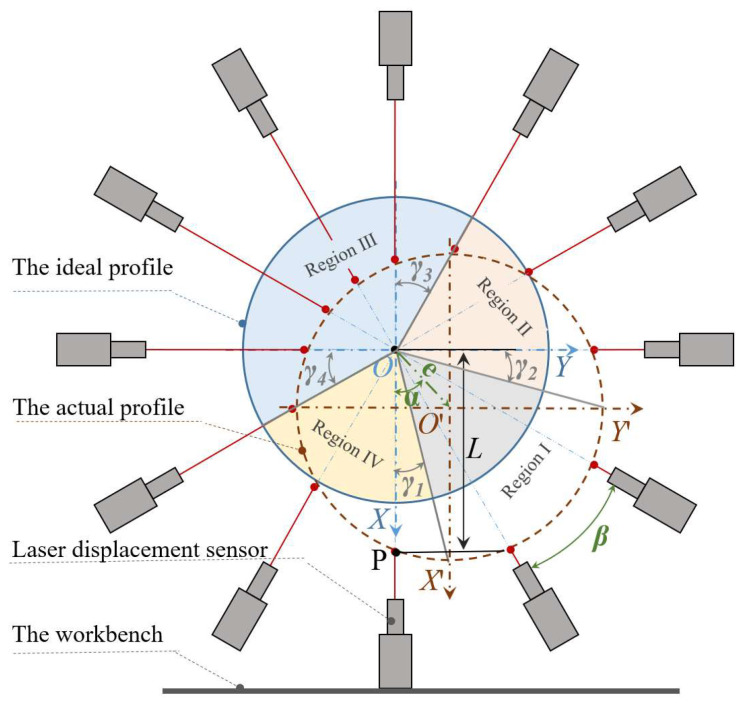
Schematic diagram of the reversal method.

Firstly, the laser displacement sensor is fixed to the grinding machine table so that the laser beam from the laser displacement sensor passes through the center of the grinding machine spindle. Secondly, the grinding wheel contour data is collected using the laser displacement sensor after starting the grinding machine spindle. Finally, the actual circle contour is reduced by polar coordinates in combination with the angular velocity of spindle rotation. As shown in Figure 1, an arbitrary point P(*x_p_*,*y_p_*) on the actual circle circumference can be expressed by Equation (1):

(1) $$x_{p}=L*\;cos\beta ;y_{p}=L*\;sin\beta$$

where *L* denotes the distance of the laser displacement sensor from the center of the spindle, and *β* denotes the radian between the two sampling points.

To evaluate the error caused by sampling unevenness, the actual contour is divided into four quadrants according to the coordinate axes, and the total number of samples of the actual contour is defined as N. The number of samples of the arc segments in the four quadrants corresponding to the actual contour is *n*_1_, *n*_2_, *n*_3_*,* and *n*_4_. Additionally, the maximum of *n*_1_, *n*_2_, *n*_3_, and *n*_4_ is defined as as *n*_max_, and the minimum as *n*_min_. The ratio ε between *n*_max_ and *n*_min_ is the evaluation of sampling uniformity, as shown in Equation (2). If ε is close to 1, it indicates that the sampling uniformity is better.

(2) $$\begin{array}{c} \Delta x=e\cdot cos\alpha ,\;\Delta y=e\cdot sin\alpha ;\\ \gamma_{1}=arctan\left(\frac{\Delta y}{R+\Delta x}\right),\;\gamma_{2}=arctan\left(\frac{\Delta x}{R+\Delta y}\right);\\ \gamma_{3}=arctan\left(\frac{\Delta y}{R-\Delta x}\right),\gamma_{4}=arctan\left(\frac{\Delta x}{R-\Delta y}\right);\\ n_{1}=\frac{\frac{\pi }{2}-\gamma_{1}-\gamma_{2}}{2*\pi }*N,n_{2}=\frac{\frac{\pi }{2}+\gamma_{2}-\gamma_{3}}{2*\pi }*N;\\ n_{3}=\frac{\frac{\pi }{2}+\gamma_{3}+\gamma_{4}}{2*\pi }*N,n_{4}=\frac{\frac{\pi }{2}-\gamma_{4}+\gamma_{1}}{2*\pi }*N\;;\\ \varepsilon =\frac{\max\left(n_{1},n_{2},n_{3},n_{4},\right)-\min\left(n_{1},n_{2},n_{3},n_{4},\right)}{\min\left(n_{1},n_{2},n_{3},n_{4},\right)} \end{array}$$

where *e* is the eccentricity value; α is the eccentric phase.

The calculation of the above Equation (2) shows that for the eccentricity level within 10 μm, the small displacement eccentricity has less influence on the spindle contour reduction, and the evaluation index ε is 1. The accuracy of the reduction contour can be guaranteed, so the reversal method can be used for the circle contour reduction.

**Figure 2:**
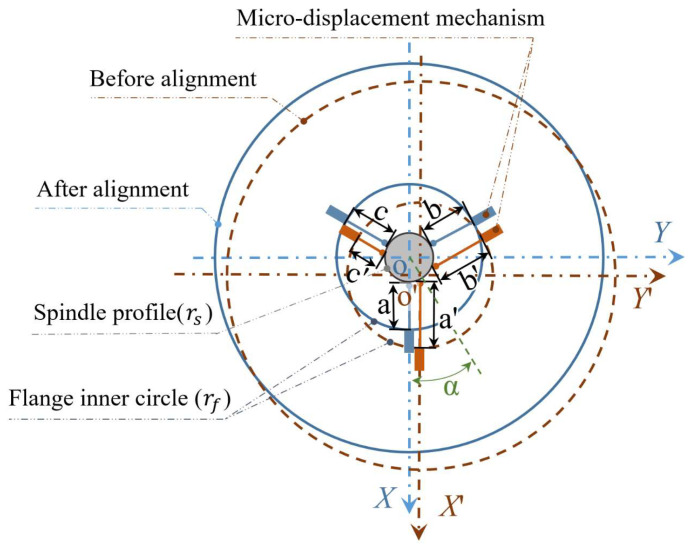
Schematic diagram of three-way alignment.

After the reconstruction of the grinding wheel contour by laser displacement sensor is verified to be feasible, a suitable micro-displacement mechanism needs to be used for fine-tuning to achieve centering. It is known from the geometry that at least three directions of micro-displacement are required to achieve in-plane centering, and the relationship between the alignment amount of the micro-displacement mechanism and the eccentricity state needs to be deduced. The *XOY* reference coordinate system is established with the center of the grinding wheel spindle as the origin of the coordinate system, as shown in Figure 2, and the right-angle coordinate system *X*′*O*′*Y*′ is established with the center of the actual grinding wheel contour, where *a′*, *b′* and *c′* are the displacement states of the micro-displacement mechanism after contacting the grinding machine spindle, and *a*, *b* and *c* are the displacement states of the micro-displacement mechanism after self-aligning. To obtain the required displacement in the three directions, the coordinates of the contact points need to be found according to the circular contour of the grinding wheel before and after alignment, respectively.

The circumferential contour before self-aligning is shown as the dashed line, and the circumferential contour after self-aligning is shown as the solid line. Defining *r* in Equation (3) is equal to the spindle radius *r_s_* and the flange inner circle radius *r_f_*, respectively. The position points of the three micro-displacement mechanisms in contact with the spindle before self-aligning can be obtained as $A_{s}^{\prime }\left(x_{as}^{\prime },y_{as}^{\prime }\right),B_{s}^{\prime }\left(x_{bs}^{\prime },y_{bs}^{\prime }\right),C_{s}^{\prime }\left(x_{cs}^{\prime },y_{cs}^{\prime }\right)$. The position points of the three micro-displacement mechanisms in contact with the inner circle of the flange before self-aligning can be obtained as $A_{f}^{\prime }\left(x_{af}^{\prime },y_{af}^{\prime }\right),\;B_{f}^{\prime }\left(x_{bf}^{\prime },y_{bf}^{\prime }\right),\;C_{f}^{\prime }\left(x_{cf}^{\prime },y_{cf}^{\prime }\right)$. The length of the three micro-displacement mechanisms before self-aligning is $a^{\prime }=\sqrt{{\left(x_{as}^{\prime }-x_{af}^{\prime }\right)}^{2}+{\left(y_{as}^{\prime }-y_{af}^{\prime }\right)}^{2}},\;b^{\prime }=\sqrt{{\left(x_{bs}^{\prime }-x_{bf}^{\prime }\right)}^{2}+{\left(y_{bs}^{\prime }-y_{bf}^{\prime }\right)}^{2}}$, $c^{\prime }=\sqrt{{\left(x_{cs}^{\prime }-x_{cf}^{\prime }\right)}^{2}+{\left(y_{cs}^{\prime }-y_{cf}^{\prime }\right)}^{2}}$.

(3) $$\begin{array}{c} \{\begin{array}{c} {\left(x_{a^{\prime }}-e\cdot cos\alpha \right)}^{2}+{\left(y_{a^{\prime }}-e\cdot sin\alpha \right)}^{2}=r^{2}\\ y_{a^{\prime }g}=e\cdot sin\alpha \\ x>0 \end{array};\\ \{\begin{array}{c} {\left(x_{b^{\prime }}-e\cdot cos\alpha \right)}^{2}+{\left(y_{b^{\prime }}-e\cdot sin\alpha \right)}^{2}=r^{2}\\ y_{b^{\prime }}=-\sqrt{3}x_{b^{\prime }}+e\cdot sin\alpha +\sqrt{3}e\cdot cos\alpha \\ x_{b^{\prime }}<0 \end{array};\\ \{\begin{array}{c} {\left(x_{c^{\prime }}-e\cdot cos\alpha \right)}^{2}+{\left(y_{c^{\prime }}-e\cdot sin\alpha \right)}^{2}=r^{2}\\ y_{c^{\prime }}=\sqrt{3}x_{c^{\prime }}+e\cdot sin\alpha -\sqrt{3}e\cdot cos\alpha \\ x<0 \end{array}; \end{array}$$

Defining *r* in Equation (4) as equal to the spindle radius *r_s_* and the flange inner circle radius *r_f_*, respectively. The position points of the three micro-displacement mechanisms in contact with the spindle after self-aligning can be obtained as *A_s_*(*x_as_*,*y_as_*), *B_s_*(*x_bs_*,*y_bs_*), *C_s_*(*x_cs_*,*y_cs_*). The position points of the three micro-displacement mechanisms in contact with the inner circle of the flange after self-aligning can be obtained as *A_f_*(*x_af_*,*y_af_*), *B_f_*(*x_bf_*,*y_bf_*), *C_f_*(*x_cf_*,*y_cf_*). The length of the three micro-displacement mechanisms after self-aligning is $a=\sqrt{{\left(x_{as}-x_{af}\right)}^{2}+{\left(y_{as}-y_{af}\right)}^{2}}$, $b^{\prime }=\sqrt{{\left(x_{bs}^{\prime }-x_{bf}^{\prime }\right)}^{2}+{\left(y_{bs}^{\prime }-y_{bf}^{\prime }\right)}^{2}},\;c^{\prime }=\sqrt{{\left(x_{cs}^{\prime }-x_{cf}^{\prime }\right)}^{2}+{\left(y_{cs}^{\prime }-y_{cf}^{\prime }\right)}^{2}}$.

(4) $$\{\begin{array}{c} x_{a}{}^{2}+y_{a}{}^{2}=r^{2}\\ y_{a}=0\\ x_{a}>0 \end{array};\;\;\;\{\begin{array}{c} x_{b}{}^{2}+y_{b}{}^{2}=r^{2}\\ y_{b}=-\sqrt{3}x_{b}\\ x_{b}<0 \end{array};\;\;\;\{\begin{array}{c} x_{c}{}^{2}+y_{c}{}^{2}=r^{2}\\ y_{c}=\sqrt{3}x_{c}\\ x_{c}<0 \end{array}$$

Therefore, to achieve self-aligning, three micro-displacement mechanisms are needed to move the distances $L_{a}=a^{\prime }-a$,$\;L_{b}=b^{\prime }-b$,$\;L_{c}=c^{\prime }-c$, with positive values representing retraction and negative values representing extension.

## 3. Structural Design of Self-Aligning Flange

Mounting an offline dressed grinding wheel on the grinding machine spindle causes circumferential runout, which requires the use of suitable alignment elements for alignment. From the above self-aligning principle, it is clear that self-aligning requires three micro-displacement output devices

The mechanisms that produce micro-displacement mainly include mechanical drive micro-displacement, linear motor micro-displacement [11], the magnetostrictive micro-displacement mechanism [12,13], and piezoelectric actuated micro-displacement [14,15]. Among them, the mechanical transmission-type displacement mechanism can produce a large stroke, but it is easy to produce backlash and friction wear and crawl phenomenon, so the accuracy is not high. Linear motor-type micro displacement intermediates without a transmission mechanism, and has a high transmission efficiency, but due to its higher cost and it being easy to heat, it is not suitable for application with the self-aligning of grinding wheel in this paper. The magnetostrictive type can produce accurate micro-displacement with better repeatability, but it is easy to heat up under the action of the magnetic field, which has some influence on its accuracy. The piezoelectric actuation type is based on the inverse piezoelectric effect, and the value of displacement is changed by adjusting the input voltage value of the piezoelectric element, which has the advantages of small size, high resolution, and high output force.

**Figure 3:**
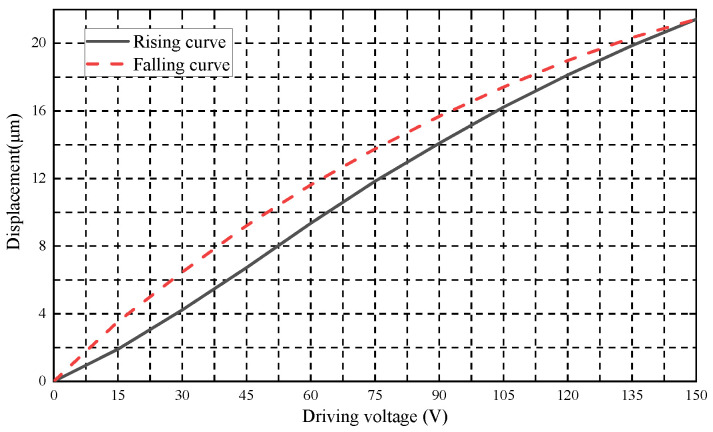
Piezoelectric characteristic curve.

Limited by the compact structure of the grinding wheel flange, the piezoelectric actuator was finally selected as the micro-displacement structure. Its piezoelectric characteristic curve is shown in Figure 3. When installing the self-aligning flange, to avoid collision between the grinding wheel spindle and the piezoelectric actuator, the piezoelectric actuator needs to be set back along the axial direction, and after the flange is installed, the piezoelectric actuator installation is moved forward along the respective axial direction until it touches the outer surface of the grinding wheel spindle.

**Figure 4:**
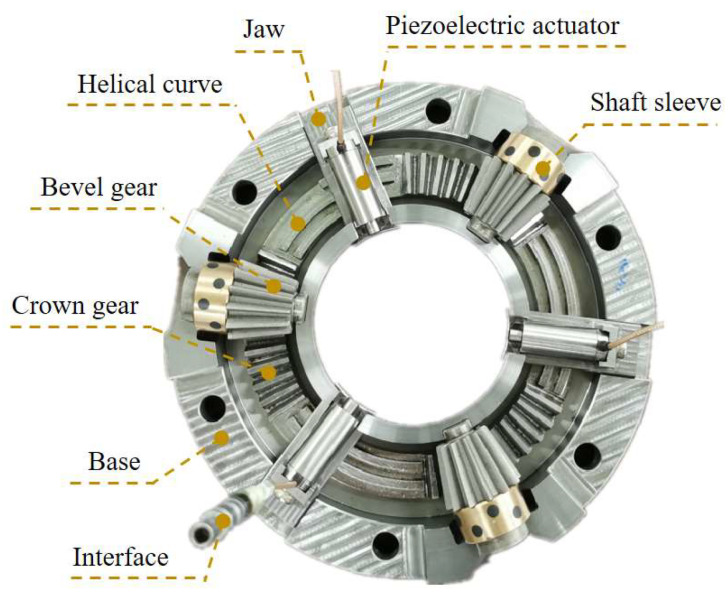
Internal diagram of self-aligning flange.

To realize the requirement of three piezoelectric actuators advancing and retreating simultaneously in their respective axial directions, the flange is designed with a three-jaws chuck structure on the lathe. As shown in Figure 4, the internal structure of the self-aligning flange consists of three jaws, three piezoelectric actuators, three bevel gears, three bushings, and a crown gear. In order to make the crown gear and bevel gears and jaws fit at the same time, it is designed to be divided into six equal parts, with the bevel tooth part alternating with the helix curve. When using the flange, a wrench is used to rotate the bevel gear and drive the crown gear to turn, thus driving the jaws to move in the respective axial direction in translation.

By attaching the piezoelectric actuator to the jaws, it can be seen that the piezoelectric actuator only touches the grinding machine spindle during alignment. It should be noted that due to the limitation of the machining accuracy of the bevel gear, there will be a backlash in the transmission, but since the self-aligning flange only uses a single reverse rotation to drive the tooth discs during use, the backlash between the bevel gear and the crown gear has no effect on its performance.

## 4. Results and Discussion

### 4.1. Processing of Circumferential Data

In sampling the grinding wheel circumference data, the displacement signal from the laser displacement sensor is theoretically started while the grinding wheel is rotating, but there is no trigger to turn on the laser displacement sensor for contour acquisition while the grinding machine spindle is rotating. Therefore, when the circular contour acquisition is performed, the laser displacement sensor is turned on first for data acquisition, and the grinding machine spindle is subsequently rotated. The contour signal is obtained as the initial segment is the same and is programmable for identification and deletion. Since the contour data has different degrees of noise, which affect the reduction of the grinding wheel circumferential contour, the original contour data needs to be noise-reduced to obtain a relatively accurate circumferential contour for the subsequent calculation of the eccentric phase and eccentricity. In this paper, the moving average filter is selected for noise reduction. The algorithm itself is based on the principle of low-pass filtering, and a total of five values are taken before and after each value as well as itself for averaging, which is a simple fast and accurate calculation principle [16,17,18].

**Figure 5:**
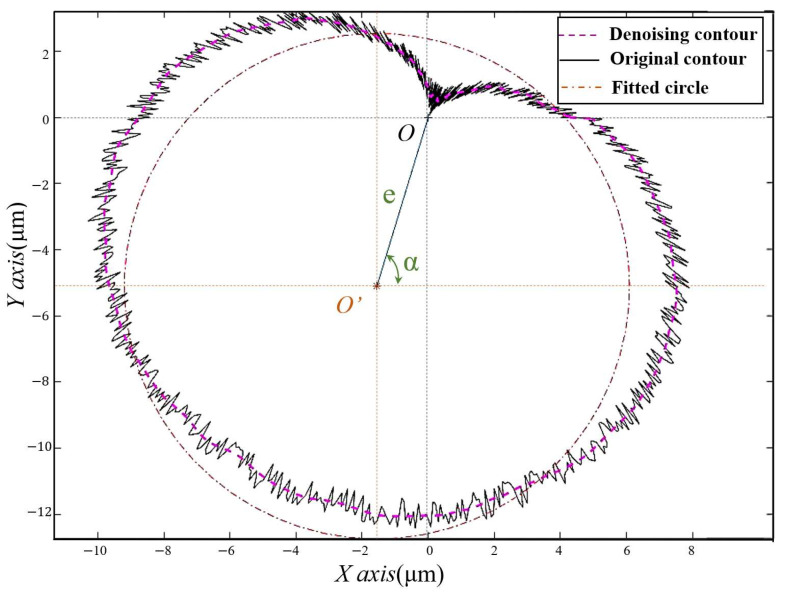
Graph of circumference data after processing.

After noise reduction of the circular signal, it is necessary to calculate the discrete points for circle fitting to obtain the eccentricity and eccentric phase. Since there are coordinates for each data point, it is theoretically possible to obtain the circle center coordinates by averaging the x and y values of all coordinates—but due to the noise and uneven distribution of sampling points in the actual sampling process, it is not possible to use this method to obtain the circle center coordinates. In this paper, the least squares method was used to fit the circle by calculating the difference between the square of the distance from the center of the circle to each data point and the square of the radius, to get the smallest difference and obtain the best-fitting circle. As shown in Figure 5, the solid line is the original data, the dashed line is the data after noise reduction; the dotted line is the fitted circle.

### 4.2. The Self-Aligning Validation Experiment

To verify the accuracy of the results of the self-aligning method and data collection and analysis, a verification experiment was conducted. In the experiment, there was no grinding wheel with a well-dressed surface available for the experiment due to the lack of an offline dressing machine for grinding wheels. Since the grinding wheel is always mounted on the self-aligning flange during the dressing and use of the grinding wheel, the eccentric characteristics presented by the grinding wheel contour data were consistent with those presented by the self-aligning flange, so the contour data collected in the verification experiment could be used as the contour data of the mating surface of the flange and the grinding wheel.

During the experiment, the laser displacement sensor was first mounted on the grinding machine table, and the grinding machine spindle was moved to find the lowest point of the spindle so that the laser beam of the laser displacement sensor could pass through the center of the grinding machine spindle circle as close as possible to the theoretical requirements. The self-aligning flange was then mounted to the grinding machine spindle and half-tightened with a nut, i.e., the self-aligning flange was fixed but the relative position could be adjusted. The self-aligning flange was moved to the recognizable range of the laser displacement sensor to start sampling, then the grinding machine spindle was turned on to rotate at low speed, and the secondary development software automatically processed the data and generated the required voltage values for the three piezoelectric actuators when the sampling was completed.

**Figure 6:**
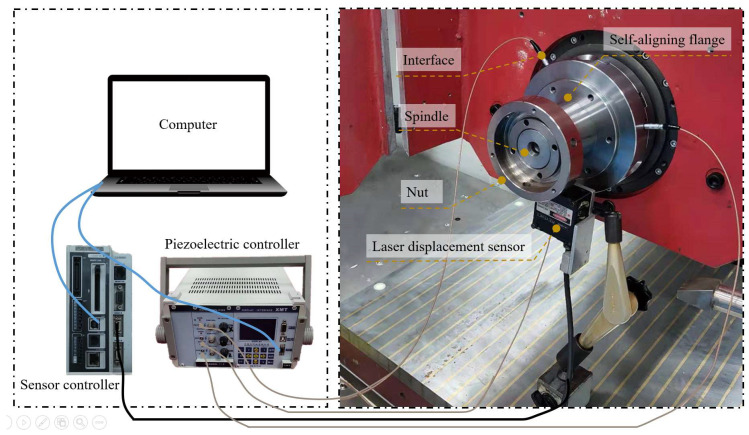
Diagram of the experimental setup.

The self-aligning process was then carried out by rotating the bevel gear with a wrench to bring the piezoelectric actuator into contact with the grinding spindle surface. The piezoelectric controller was connected to the computer, and a signal cable was used to connect the piezoelectric actuator to the piezoelectric controller, as shown in Figure 6. After the connection was made, the secondary development software sent the calculated voltage value to the piezoelectric controller, and the output voltage value from the piezoelectric controller caused the piezoelectric actuator to move accordingly. When the piezoelectric actuator was displaced for self-centering, the nut was fully tightened. The signal line connected to the piezoelectric actuator was then removed, and the centered circumferential contour was collected and compared. Detailed experimental parameters are shown in Table 1.

**Figure 7:**
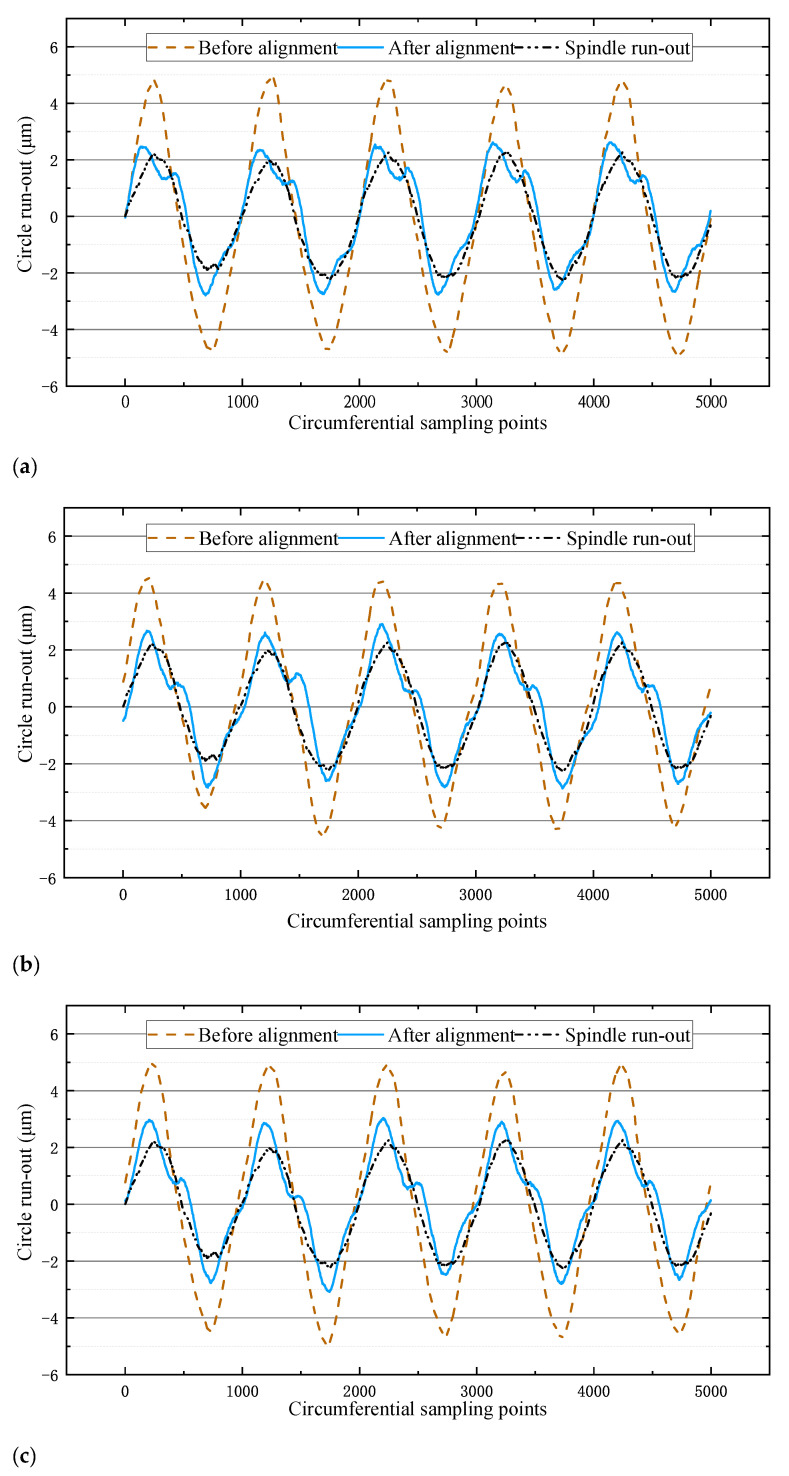

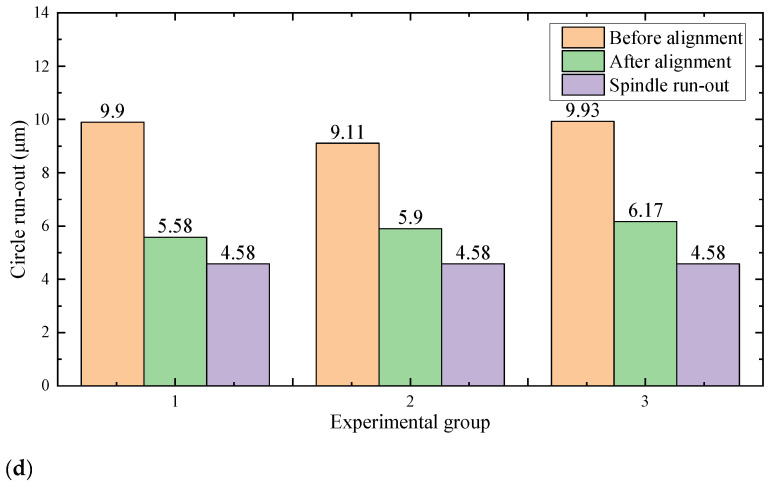
Experimental results. (**a**) Results of the first self-aligning experiment. (**b**) Results of the second self-aligning experiment. (**c**) Results of the third self-aligning experiment. (**d**) Comparison chart of the three experimental results.

After self-aligning according to the calculated theoretical voltage value, it was found that the circumferential runout value did not change significantly. As the piezoelectric characteristic curve in Figure 3 was obtained under non-stressed conditions, the piezoelectric actuator did not get the ideal displacement under stressed conditions by inputting the theoretical voltage, so the subsequent experiment could be centered according to the theoretical eccentric phase obtained by the algorithm. Through several attempts, self-aligning could be achieved by giving the piezoelectric actuator 0.8 times its maximum voltage input. Accordingly, three repetitive experiments were carried out respectively, and the results of the centering were obtained as shown in Figure 7.

From the experimental results in Figure 7, it can be seen that the average eccentricity before the alignment of the three experiments was 9.65 μm, and the average eccentricity after the alignment was 5.88 μm. Since the eccentricity of the spindle itself was 4.58 μm as measured by the laser displacement sensor, it can be calculated that the eccentricity of the flange was reduced by 74% after the self-aligning, which can meet the production requirements of the grinding machine.

During the self-aligning experiment, the grinding machine spindle and flange had a tapered fit, which itself had a good centering effect [19]. Therefore, in the process of half-tightening and full-tightening of the nut, there is a possibility that the full-tightening will lead to a fuller conical fit, resulting in the centering effect. In addition, due to the use of the three-jaws chuck structure for the movement of the piezoelectric actuator, the self-aligning flange may be aligned due to the centering function of the three-jaws chuck [20]. In order to investigate the cause of the self-aligning phenomenon more accurately, the experiment of nut tightness and the single-factor experiment of three-jaws centering were carried out. The experimental parameters are shown in Table 2 and Table 3.

**Figure 8:**
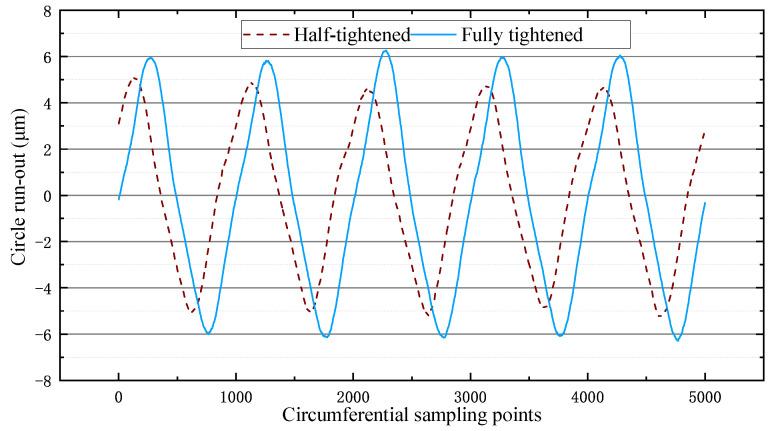
Graph of nut tightness experimental results.

From the results of Figure 8, it can be seen that the eccentricity was 10.32 μm at half-tightening and 12.56 μm at full tightening, so the tightening of the nut did not affect the self-aligning of the flange.

**Figure 9:**
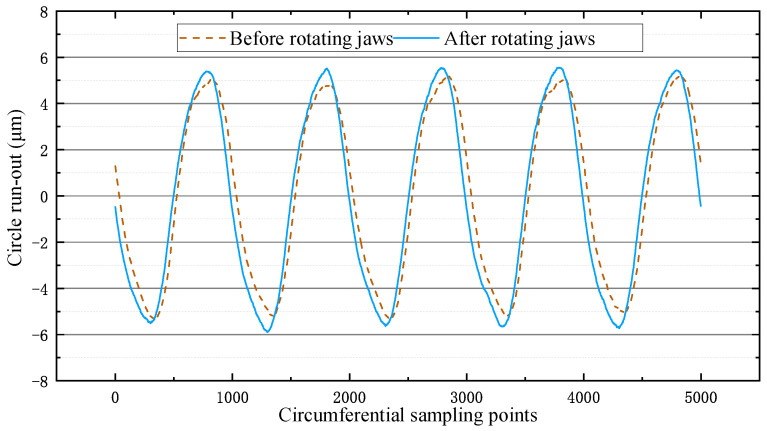
Graph of rotating jaws experimental results.

From the results in Figure 9, it can be seen that the eccentricity before the spin jaws was 10.53 μm and the eccentricity after the spin jaws was 11.44 μm, both of which are basically the same. Therefore, using the jaws alone to hold the spindle did not result in self-aligning. In summary, the self-aligning flange designed in this paper can realize the self-aligning function through the expansion and contraction of the piezoelectric actuator and has a higher alignment efficiency than the results of Chen et al.

## 5. Conclusions

The problem of eccentricity exists when the dressed grinding wheel is mounted to the grinding machine spindle, and the traditional manual alignment requires several alignments, which is less efficient and less accurate. In this paper, a self-aligning flange based on a piezoelectric actuator is proposed to replace the traditional manual alignment of the grinding wheel, and the eccentricity is reduced by 74% after the self-aligning. This greatly ensures the accuracy of the grinding process and reduces the damage to the grinding machine due to grinding wheel vibration. The self-aligning flange significantly reduces the time needed for grinding wheel installation and has a good engineering application prospect. The main innovations of this paper are as follows:

(1) The uneven error in the reduction of the grinding wheel contour using the reversal method was investigated.
(2) Based on the dimensions of the universal flange, bevel gears, jaws, and tooth discs were designed to control the movement of the piezoelectric actuator.
(3) The eccentricity and eccentric phase were obtained by processing the raw data with averaging filters and using the least squares method for circle fitting.
(4) The laser displacement sensor acquisition and the piezoelectric controller were combined by secondary development software to shorten the operation time and improve the efficiency of grinding wheel installation and centering.

## Figures and Tables

**Table 1:** Experimental parameters.

Spindle Speed (rpm)	Sampling Frequency (Hz)	Number of Sampling Laps	Diameter of Flange Mating Surface (mm)
60	1000	5	74

**Table 2:** Single-factor experimental parameters of nut tightness.

Spindle Speed (rpm)	Sampling Frequency (Hz)	Number of Sampling Laps	Diameter of Flange Mating Surface (mm)	Tightness of Nut
60	1000	5	74	Half-tightened/Fully tightened

**Table 3:** Single-factor experiment of three-jaws aligning.

Spindle Speed (rpm)	Sampling Frequency (Hz)	Number of Sampling Laps	Diameter of Flange Mating Surface (mm)	Experimental Factors
60	1000	5	74	With jaws/Without jaws
